# Chromosome Division in Early Embryos—Is Everything under Control? And Is the Cell Size Important?

**DOI:** 10.3390/ijms25042101

**Published:** 2024-02-09

**Authors:** Adela Horakova, Marketa Konecna, Martin Anger

**Affiliations:** 1 Department of Genetics and Reproductive Biotechnologies, Veterinary Research Institute, 621 00 Brno, Czech Republic; 2 Institute of Animal Physiology and Genetics, Czech Academy of Science, 277 21 Libechov, Czech Republic; 3 Faculty of Science, Masaryk University, 602 00 Brno, Czech Republic

**Keywords:** spindle, chromosome division, segregation errors, spindle assembly checkpoint, embryo, CDK1, cell size, aneuploidy

## Abstract

Chromosome segregation in female germ cells and early embryonic blastomeres is known to be highly prone to errors. The resulting aneuploidy is therefore the most frequent cause of termination of early development and embryo loss in mammals. And in specific cases, when the aneuploidy is actually compatible with embryonic and fetal development, it leads to severe developmental disorders. The main surveillance mechanism, which is essential for the fidelity of chromosome segregation, is the Spindle Assembly Checkpoint (SAC). And although all eukaryotic cells carry genes required for SAC, it is not clear whether this pathway is active in all cell types, including blastomeres of early embryos. In this review, we will summarize and discuss the recent progress in our understanding of the mechanisms controlling chromosome segregation and how they might work in embryos and mammalian embryos in particular. Our conclusion from the current literature is that the early mammalian embryos show limited capabilities to react to chromosome segregation defects, which might, at least partially, explain the widespread problem of aneuploidy during the early development in mammals.

## 1. The Assembly of the Spindle Is under Surveillance

Cell cycle is an extremely complex activity used by cells to create a new generation. It requires the precise spatial and temporal coordination of multiple events. The central role in controlling cell cycle progression is played by kinases, namely Cyclin-dependent kinases (CDKs) and opposing phosphatases [1,2]. Protein phosphorylation also provides an important temporal and spatial coordination of cell cycle events [3]. The crucial objective during the cell cycle is to preserve the integrity of the genetic information transferred to the daughter cells. From this perspective, the two events, which seem particularly risky, are the replication of chromosomes during the S phase and their segregation during the M phase. Because preservation of the genomic integrity is essential to their survival, cells have evolved surveillance mechanisms that allow cell cycle progression only when the preceding events have been correctly completed. Such mechanisms are called checkpoints, and they control most of the important cell cycle transitions, such as entry into S phase, entry into mitosis, mitotic spindle assembly, and cytokinesis. In this review, we will focus on the mechanisms controlling the assembly of the spindle apparatus. The spindle is a dynamic machine that is built upon entry into mitosis, and which then ensures accurate chromosome segregation during anaphase. The monitoring mechanism associated with its assembly is called SAC. Our aim is to discuss the importance of this mechanism for the development of early embryos, particularly in mammals.

The essential organelle for the assembly of the spindle in somatic cells are centrosomes [4,5]. The first steps of spindle assembly are initiated in G2 before nuclear envelope breakdown (NEBD) and involve the positioning of both centrosomes at the opposite side of the nucleus [6,7]. After NEBD, the centrosomes produce microtubule bundles, which interact with kinetochores, as well as astral microtubules, important for positioning the spindle within the cell [8]. In animal cells, the spindle assembly requires both centrosome and chromatin-mediated microtubule nucleation. Unlike somatic cells or spermatocytes, mammalian oocytes do not possess the centrosomes and the spindle is assembled using simplified microtubule-organizing centers (MTOCs), which are activated in the vicinity of the chromatin [9,10,11,12,13]. The meiotic spindles also lack astral microtubules and rely on the actin network for their positioning close to the cortex before anaphase, and the actin–microtubule interactions are also crucial for correct chromosome segregation [14,15,16,17,18,19,20]. The embryonic development after fertilization is characterized by a transition from MTOC to a centrosome-driven spindle, and the timing of this transition is species dependent. Whereas in mice, the sperm centrioles are lost during spermiogenesis [21,22], and the transition from MTOC to a centrosome-based bipolar spindle is gradual in early embryos [23,24,25]; in non-rodent mammalian embryos, such as human, it seems that the sperm centriole is utilized for spindle assembly in later stages [26,27,28]. However, when the human embryos start utilizing centrosomes from the sperm still remains poorly understood, and a recent study with cattle showed that centrioles might be assembled de novo [29].

**Figure 1:**
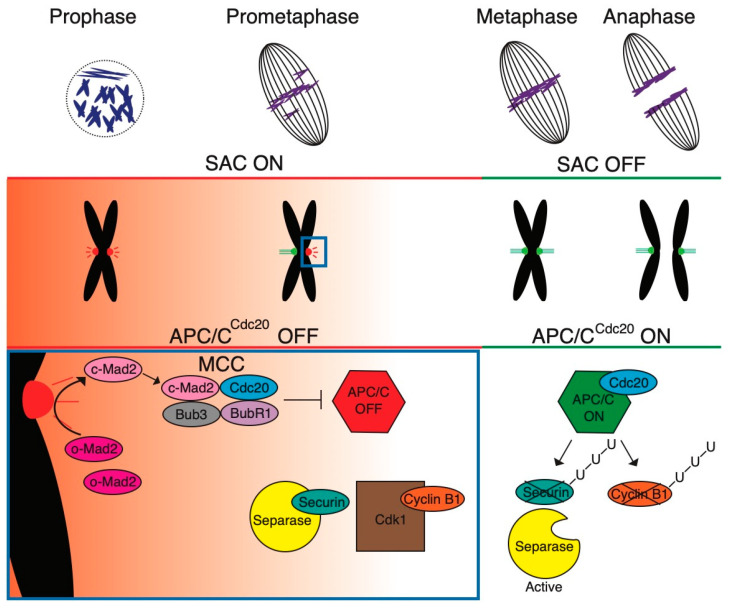
Control of spindle assembly by SAC. During cell division, chromosomes interact with the spindle microtubules via kinetochores, large protein complexes attached to the centromeric chromatin. The Spindle Assembly Checkpoint (SAC) is triggered during prometaphase in response to unattached, or improperly attached, kinetochores. The activated SAC assembles the mitotic checkpoint complex (MCC) consisting of mitotic arrest deficient 2 (Mad2), budding uninhibited by benzimidazole 3 (Bub3), budding uninhibited by benzimidazole-related 1 (BubR1), and cell division cycle 20 (Cdc20) proteins. Mitotic arrest deficient 1 (Mad1)/closed Mad2 (c-Mad2) complex recruits and converts cytosolic open Mad2 (o-Mad2) into c-Mad2, which is associated with Bub3, BubR1, and Cdc20 mentioned above. The MCC inhibits the Anaphase Promoting Complex/Cyclosome (APC/C), preventing Securin and Cyclin B destruction, thus inducing the mitotic arrest. The SAC remains active until all kinetochores have established stable microtubule connections, at which point it becomes inactive. After that, the SAC is deactivated through MCC disassembly. Subsequently, Cdc20 binds and activates APC/C, initiating ubiquitination (U) of Cyclin B and Securin. This leads into cohesin cleavage and anaphase. Concurrently, cyclin-dependent kinase 1 (CDK1) inactivation facilitates exit from mitosis (based on [33]).

Since the separation of sister chromatids is an irreversible step, the errors during spindle assembly, specifically errors in the connection of kinetochores to microtubule bundles, have potentially severe consequences. If uncorrected, the daughter cells might suffer from numerical chromosomal aberrations, also called aneuploidy. Such a situation leads to cell death, or in the case of embryos, to the termination of development. Aneuploidy is also very frequent in cancer cells [30,31,32], although sometimes it is not clear whether it is a cause or a consequence. To prevent errors during chromosome segregation, cells evolved SAC, which is also called the mitotic checkpoint [32,33,34]. The SAC activity starts upon entry into mitosis, since some of the SAC players, namely protein mitotic arrest deficient 1 (Mad1), are before NEBD sequestered from chromosomes by the nuclear membrane [35]. The SAC activity then continues throughout mitosis, until the assembly of the spindle is completed, which is when all kinetochores are properly connected to the spindle apparatus. Then, the SAC activity ceases, and the cell is ready to initiate anaphase. As a direct consequence of termination of SAC activity, E3 ubiquitin ligase called Anaphase Promoting Complex/Cyclosome (APC/C) is activated and drives the cell from metaphase to anaphase [36,37]. APC/C targets multiple proteins that were important for metaphase, including Securin and Cyclin B, by their ubiquitination to destruction at the proteasome [35]. The decline of cyclin-dependent kinase 1 (CDK1) activity and destruction of Securin activates Separase, which then cleaves the sister chromatid cohesion protein 1 (Scc1) or meiotic recombination protein Rec 8 (Rec8) subunit of the cohesin complex holding together sister chromatids, initiating poleward movement of the sister chromatids (Figure 1).

The identification of the proteins required for SAC was instrumental to the understanding of its core mechanism [38,39]. The complexes formed by these proteins are essential for the monitoring of the attachment of the kinetochores to spindle microtubules, as well as for postponing the APC/C activity. The kinetochores, assembled on centromeres, specifically their outer layer, are essential for microtubule capture, and this process is specifically dependent on kinetochore scaffold 1 (KNL1), MIS12 kinetochore complex component (MIS12), and NDC80 kinetochore complex component (NDC80) KNL1-MIS12-NDC80 complex (KMN) network [40]. The KMN also participates during the interaction of SAC proteins with kinetochores [41]. Also, monopolar spindle 1 kinase (Mps1) is essential for the recruitment of SAC proteins [42,43,44]. The unoccupied kinetochore then serves as a catalytic platform for assembling the Mitotic Checkpoint Complex (MCC) [45,46]. MCC assembly first requires the KMN-dependent loading of the Mad1/mitotic arrest deficient 2 (Mad2) complex to the unattached kinetochore [47]. Subsequently, the kinetochore-loaded Mad2 protein undergoes conformational change from open (o-Mad2) to closed form (c-Mad2), which allows its binding to cell division cycle 20 (Cdc20) upon transition from the kinetochore to the cytoplasm [48,49,50]. Cdc20 is a coactivator of APC/C and until the c-Mad2 is produced by the unattached kinetochore, Cdc20 is bound to MCC and APC/C remains inactive. Each MCC complex is composed of c-Mad2, budding uninhibited by benzimidazole-related 1 (BubR1), and budding uninhibited by benzimidazole 3 (Bub3), and two Cdc20 molecules [51]. The MCC prevents APC/C activation in several ways. Well known is the sequestration of Cdc20 to the complex, which makes it inaccessible for APC/C, but it also interacts directly with APC/C [33]. The MCC is produced until all kinetochores within the cell are captured by microtubule bundles. And until this is achieved, the activation of APC/C is delayed. For its desired sensitivity, SAC must be able to detect small quantitative changes in kinetochore occupancy. It seems that SAC, at least in somatic cells, is sufficiently sensitive and able to respond to the number of microtubules attached at each specific kinetochore [52,53,54]. SAC function is however yet to be studied in species, which have a large number of chromosomes, and where the single unoccupied kinetochore would produce a proportionally lower signal. The inactivation of SAC signaling is initiated by the microtubule end binding to a kinetochore, which interrupts the assembly of SAC subunits on the kinetochore and terminates the MCC production. For complete SAC inactivation, it is however important to also dismantle the existing cytoplasmic MCC, as well as MCC bound to APC/C, and prevent its rebinding [34]. The crucial step in MCC inactivation is the thyroid hormone receptor interactor 13 (TRIP13) and p31comet-dependent conformational change of Mad2 back to its open form [55].

Another aspect equally important for faithful chromosome segregation is the orientation of sister chromatids to the spindle poles [56]. The correct orientation requires the sister kinetochores to face the opposite spindle poles. Such an orientation is called bipolar, or amphitelic attachment, and there is a higher chance of accurate chromosome segregation during anaphase. Improper attachment, either when both sister kinetochores are connected to the same pole of the spindle (synthelic attachment), when one is not connected at all (monotelic attachment), or when one kinetochore is connected to both poles (merotelic attachment), usually leads to the activation of SAC and the correction mechanisms [56]. The merotelic attachment represents a serious problem, since it escapes the SAC detection [57,58]. In meiosis I, the homologous chromosomes are connected via cohesin, located distally to the chiasmata, and sister kinetochores act as a unit being oriented to the same spindle pole [59]. The tension required for the stabilization of kinetochore attachment is transferred via chiasmata to homologous kinetochores [60]. The SAC in meiosis I is, however, blind to univalent chromosomes, because they are capable to turn off the SAC by bipolar attachment to the spindle apparatus [61,62]. The correction mechanisms, which are activated on unstable or erroneous microtubule kinetochore connections, require the activity of chromosomal passenger complex (CPC) and, specifically, Aurora B kinase, which disengage the incorrect connections by phosphorylation of the kinetochore components [63,64]. The correction mechanisms are active, even after SAC is turned off [65,66,67], which provides the opportunity for the correction of improper attachments even during anaphase.

## 2. Important Aspects of Early Embryonic Development with Potential Impact on Chromosome Segregation

Early embryonic development is initiated by the fusion of oocyte and sperm, which are both highly differentiated cells. This leads to the formation of a single-cell embryo called zygote [68]. The sperm and the egg are not fully synchronized in cell cycle during their fusion. Whereas the sperm has already completed both meiotic divisions and has a haploid number of chromosomes and protamine-like proteins largely replacing histones [69], the egg is arrested in metaphase II, with an intact spindle. Sperm contributes to the fertilization clearly by DNA, but also brings an important molecule that is called phospholipase C zeta (PLCζ), which is capable to abrogate the metaphase II block of the egg [70]. This molecule triggers a signaling cascade, releasing calcium from the deposits in the smooth endoplasmic reticulum. This leads to APC/C activation, the degradation of Cyclin B, and consequently into anaphase and the second polar body extrusion.

After fertilization, the zygote forms two pronuclei, which in some species, for example in mouse, differ in size. The pronuclei migrate towards each other, and when they are in close proximity, they undergo NEBD. During their migration, both pronuclei execute the first round of DNA replication after fertilization. The mitotic spindle in the zygote is unique; it is formed by a junction of two separate spindles that first assemble individually around each set of parental chromosomes [71]. The complex spindle assembly is perhaps a reason why the first mitosis is longer than the following mitoses in developing embryos [72]. Subsequent cell cycles of the embryo are characterized by a series of rapid divisions, called cleavage cycles, during which cells divide without growth. Unlike Drosophila, Xenopus and *C. elegans* embryos, which initially lack G1 and G2 phases [73,74,75], in mouse embryos, the gap phases are present, albeit they are significantly shortened [76,77]. During the early embryonic development, lasting in mammals around 4–8 days depending on the species, embryos move through the oviduct towards the uterus.

The onset of embryonic development is unique with respect to gene expression because of the silencing of genomic transcription [78,79]. The silencing starts in fully-grown oocytes and resumes after fertilization, in the 2-cell stage in mice or in 8–16 cell stage in humans and cattle. Therefore, the two meiotic divisions, fertilization, and one-to-three cell cycles of the embryo in mammals are executed without genomic transcription, and all the cellular activities are achieved by regulated translation instead [79]. As mentioned before, the spindles in mammalian cleavage-stage embryos are formed, at least initially, from MTOCs since the centrosomes are missing [13,27]. Such spindle assembly seems, at least in oocytes, to be prone to errors [80]. And the data from mouse oocytes also show that MTOCs-based spindles are sensitive to in vitro culture [81]. It is therefore conceivable that the MTOC spindle assembly in early embryos might represent, similarly to oocytes, a potential risk concerning chromosome segregation errors.

## 3. Control of Chromosome Segregation during Early Embryonic Development

Control of spindle assembly and correction of potential errors is vital, since the aneuploidy has severe consequences. In comparison to somatic cells, the frequency of aneuploidy in mammalian oocytes and embryos is extremely high, and it further increases with maternal age [82,83,84,85,86,87,88]. In fact, the aneuploidy represents the most frequent single cause of termination of embryonic development [89]. Aneuploidy arising during meiosis, or in zygote, uniformly affects all cells within the embryo. The aneuploidy of autosomes will most frequently result in embryo loss, with only specific exceptions that are compatible with embryonic and fetal development. In humans, these are the trisomies of chromosomes 21, 18 and 13, causing Down, Edwards, or Patau syndromes, respectively. And although the embryos survive, the cell behavior is changed profoundly, exhibiting phenotypes related to the presence of an extra chromosome [90,91,92].

Most frequently, however, the embryos are a mosaic, containing a mixture of euploid and aneuploid cells, since the aneuploidy originated later during development. And although the in vitro conditions increase the frequency of aneuploidy significantly [93], it affects mammalian embryos developing in vivo as well [94]. In vivo mouse embryos showed aneuploidy rate per blastomere from 4% in zygotes to 7% in 8-cell embryos, with steep increase to 11% between 8 to 16-cell embryos. The frequency of aneuploidy up to the 8-cell stage is not much different from the levels reported previously in meiosis II oocytes [95,96]. However, due to the increasing number of cells constituting the embryo, this translates into a much higher frequency of embryos carrying aneuploid blastomeres. Similar frequencies were reported in the embryos of other mammalian species, including human [97], cattle [93,98], pig [99] and rhesus monkey [100]. Experiments in which aneuploid blastomeres were traced in live developing embryos showed that the aneuploid cells within the embryo survive and proliferate, and they are not eliminated until hatching [94,101]. We can only speculate what is behind their ability to survive (and divide) for prolonged time. Is it the stockpile of maternal mRNAs and proteins inherited from the oocytes? Their survival could also indicate that the control mechanisms of chromosome segregation might be less active in early embryos. Nevertheless, in situations when the aneuploid blastomeres represent a significant fraction of cells constituting the embryos, the implantation and future development might be compromised. It all depends on the proportion between euploid and aneuploid cells. On the other hand, when the number of aneuploid cells within the embryo is not too high, the potential of such mosaic embryos to implant and develop further is similar to the euploid embryos [102,103]. It was shown that the cell cycle profile of aneuploid blastomeres within the embryo exhibits characteristic abnormalities, which can be used in non-invasive embryo screening [104]. In summary, it is obvious that the aneuploidy caused by undetected or uncorrected chromosome segregations errors represents a threat for embryonic development in mammals.

**Figure 2:**
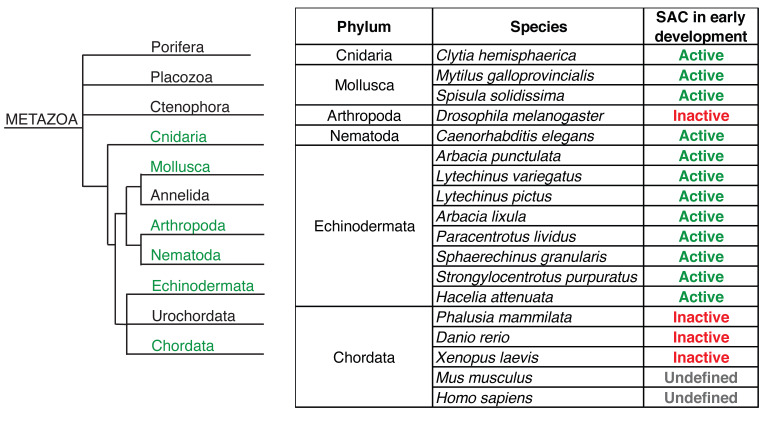
The SAC activity in Metazoans. Within the metazoan group, there are differences in SAC activation during early embryonic development. The table lists the individual representatives in which SAC activity was examined during early development; the Metazoan group is diverse in the case of SAC activity (based on [119,120]).

Phylogenetic studies focused on the presence of SAC genes in various eukaryotes showed that it is indeed a highly conserved evolutionary mechanism. It is possible to trace its origin back to a eukaryotic common ancestor whose genome already harbored multiple genes from SAC and APC/C pathways and complex structures typical for the kinetochore [105,106,107,108,109]. We also only have limited information from non-mammalian eukaryotes as to whether they utilize SAC in embryonic development (Figure 2). In *C. elegans* for example, the SAC in early embryos is functional and capable of preventing chromosome segregation errors induced by disruption of the spindle [110]. And although it shows similarities to the SAC known from mammalian cells, there are also important differences, namely in the centralization of the response [111]. And the SAC seems to be weaker in embryonic blastomeres than it is in the adult germline stem cells [112]. In general, the strength of the SAC response in *C. elegans* seems to be linked to the cell fate and cellular volume [113]. In zebrafish, on the other hand, the SAC response is detectable only after midblastula transition [114]. And its onset does not require transcription from embryonic genome and does not exhibit sensitivity to changes in nuclear-to-cytoplasmic ratio. In Xenopus, the SAC is absent in Xenopus early embryo [115] and, similarly to zebrafish, Xenopus embryos acquire sensitivity to microtubule poisons later during embryonic development in a transcription-independent manner [116]. In Xenopus oocyte extracts, however, a reaction similar to SAC can be induced by increasing the density of sperm nuclei [117]. In tunicate *Phalusia mammilata*, SAC is inactive or unresponsive until the 8th cleavage cycle, and its stringency afterwards depends on the cell size and fate [118]. And the comparison of responses to spindle disruption between various marine animals showed adequate response in sea urchins, mussels, and jellyfish and the absence of such response in ascidian and amphioxus embryos [119]. The ascidian embryos also lack the ability to accumulate the core SAC proteins, such as Mad1 and Mad2, on unattached kinetochores, further supporting the evidence that the SAC in these animals is not active in early embryos (Figure 2).

SAC, as a main mechanism controlling spindle assembly and chromosome segregation, was studied more extensively in mammalian oocytes than in the embryos. The gene knockout studies in oocytes have shown that the SAC is required for maintaining the length of meiosis I in mice and for faithful segregation of homologous chromosomes [121,122,123]. However, it was shown later that SAC is unable to postpone APC/C activation and anaphase in situations when the spindle is clearly not assembled properly and chromosomes lack congression [124,125,126,127,128,129,130]. This was similar between mice and humans, although the most frequent type of defects in spindle assembly is specific for each species. And similar insensitivity to apparent chromosomal and spindle defects was also recently reported in meiosis II mouse oocytes [84]. Thus, it seems possible that, in fact, the failure of the correction mechanisms, rather than a defect in sensing mechanisms, might contribute to frequent chromosome segregation errors in oocytes.

Although both undergo mitosis, in certain aspects of control of chromosome segregation and cell cycle progression, mammalian embryos are unique, in comparison to the somatic cells. On one hand, embryos are able to quickly achieve microtubule-to-kinetochore connections and establish chromosome congression without defects [131]. On the other hand, they seem to lack a cell-cycle timer and tend to spend longer in mitosis, which increases the probability of chromosome missegregation due to the weakening of chromosome cohesion [132]. They also frequently harbor micronuclei resulting from the encapsulation of lagging chromosomes or chromosomal fragments by membranes [100,133,134,135]. And unlike in somatic cells, the micronuclei in embryos do not contribute to chromothripsis [133].

In contrast to the oocytes, we have rather limited information about SAC function during early embryonic development in mammals. Challenging SAC using spindle depolymerizing drugs in human embryos showed that they become sensitive in later stages when they respond by apoptosis [136]. Regarding the expression of SAC core components in the embryo, it was shown that Mad2, Bub3, and BubR1 are detectable in zygotes and their depletion by RNAi caused the acceleration of the first mitosis, insensitivity to nocodazole, and chromosome segregation defects [137]. Another study, however, showed that in zygotes and two-cell embryos, the interaction of overexpressed Mad1 with kinetochores is only brief and that the protein quickly disengages from the chromosomes after NEBD [138]. Recently published results showed that mouse morulas are insensitive to chromosome segregation defects, and the misaligned chromosomes do not delay the onset of anaphase [139]. Interestingly, in this work, the authors show that a mild inhibition of APC/C by ProTame prolonged mitosis and simultaneously reduced the occurrence of micronuclei and misaligned chromosomes. The gene deletion studies, targeting SAC proteins Mad1, Mad2, budding uninhibited by benzimidazole 1 (Bub1), and BubR1, consistently reported loss of homozygous embryos around the blastocyst stage [140,141,142,143,144]. And although for most of these gene knockouts, we have no details about the consequences at the single-cell level, Mad2^-/-^embryos showed increased frequency of chromosome segregation defects and loss of sensitivity to nocodazole, which is consistent with loss of SAC. The gene deletion studies are, however, unable to distinguish whether the blastomeres of early embryos do not utilize SAC, or whether the stockpile of maternal mRNAs and proteins can initially compensate for the lack of newly transcribed mRNAs.

## 4. Is Cell Size Important for the Fidelity of Chromosome Segregation?

A remarkable difference between oocytes and embryonic blastomeres, and average somatic cells is in their size. By their volume, oocytes and blastomeres are among the biggest cells in the body. The size of the germ cell is certainly essential for species with external development, such as fish, amphibians, reptiles, birds etc., because their life depends on resources from the egg. The large size of the female gametes might not be so important in mammals, although their development depends on maternal stockpile, until they activate their genome as well. Nevertheless, the bigger cellular volume might not be always advantageous, and we might wonder whether the higher frequency of chromosome segregation errors is the price for it. The relationship between the cell size and the SAC was first studied in *C. elegans* embryos. The authors of the study noticed that the ability of nocodazole to induce cell cycle delay is proportional to the size of the cell [145]. This means that the stringency of SAC drops with increasing cellular volume. The authors concluded that in larger cells, the MCC produced by kinetochores is diluted and unable to inhibit APC/C activity in the larger cytoplasm. This was subsequently tested by manipulation of the cytoplasmic volume of mouse oocytes [146]. Using micromanipulation and fusion techniques, the authors created oocytes with reduced and increased volumes of cytoplasm. Using this procedure, the authors showed that the volume of the cytoplasm affects various parameters, including duration of meiosis I, spindle morphology, chromosome congression, and importantly, also the SAC stringency. The authors suggested that when cell volume is reduced, MCC complex is more concentrated, and this increases SAC stringency. Using a different approach and producing mouse oocytes with smaller volumes of cytoplasm, another group confirmed that the SAC stringency depends on the volume of the cytoplasm [147]. They showed that the reduction of the cytoplasm prolonged the interval between germinal vesicle breakdown (GVBD) and APC/C activation; however, they also showed that the congression and attachment defects are unable to prevent anaphase in oocytes with reduced cytoplasmic volume. It is also important to consider that in *C. elegans*, apart from the cell size, SAC strength also depends on lineage specificity [113]. The impact of cell size and lineage specificity on SAC stringency was also confirmed using ascidian *Phallusia mammillata*, in which SAC is turned on in cells only after midblastula transition [118]. The link between the cell size and SAC stringency was also recently tested in two-cell mouse embryos [137]. The results showed no difference in response to the low levels of nocodazole in intact blastomeres, or blastomeres with reduced volume, indicating that the cell size in mouse embryo is not linked to the stringency of SAC. In conclusion, in certain species, such as *C. elegans*, the link between the cell volume and the ability of SAC to postpone anaphase is very strong. In mammals, the link exists in oocytes, but it seems to be uncoupled in embryos.

## 5. Conclusions

**Figure 3:**
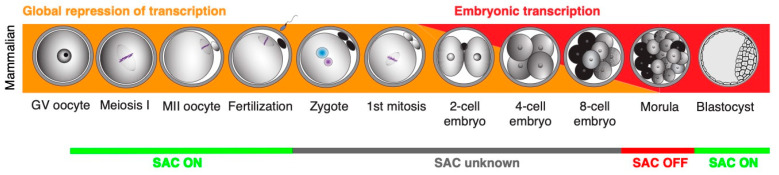
The overview of early development in mammals and SAC activity.

The published work summarized here shows clearly that our knowledge about the control mechanisms of chromosome segregation in early embryos is fairly limited (Figure 3). And considering how important a problem the aneuploidy in early embryo represents, this gap of knowledge should be addressed urgently. Studies following the expression and localization of SAC proteins in early embryonic mitoses, gene depletion studies specifically targeting the SAC components, and also functional studies challenging the SAC, would help to obtain a better picture about SAC function during early development. It is conceivable that the results might in fact reveal that, similarly to Xenopus and zebrafish, the SAC in early mammalian embryos is inactive. If this is the case, it will be important to address when its functionality is regained and, more importantly, how chromosome segregation in early embryos is controlled without SAC.

During meiosis, SAC controls the correct attachment of all chromosomes to the meiotic spindle and prevents aneuploidy. During early embryonic development, the situation is more complicated. The main problem is that we do not have reliable data on SAC function during early development in most species. From the data that we have at present, it seems that SAC regulates chromosome segregation only in later development (based on: [12,79,136,139]).

## Abbreviations

APC/C	anaphase-promoting complex/cyclosome
Bub1	budding uninhibited by benzimidazole 1
Bub3	budding uninhibited by benzimidazole 3
BubR1	budding uninhibited by benzimidazole-related 1
c-Mad2	closed Mad2
Cdc20	cell division cycle 20
CDK	cyclin-dependent kinase
CDK1	cyclin-dependent kinase 1
CPC	chromosomal passenger complex
GVBD	germinal vesicle breakdown
KMN	KNL1-MIS12-NDC80 complex
KNL1	kinetochore scaffold 1
Mad1	mitotic arrest deficient 1
Mad2	mitotic arrest deficient 2
MCC	mitotic checkpoint complex
MIS12	MIS12 kinetochore complex component
Mps1	monopolar spindle 1 kinase
MTOC	microtubule-organising centre
NDC80	NDC80 kinetochore complex component
NEBD	nuclear envelope breakdown
o-Mad2	open Mad2
PLCζ	phospholipase C zeta
Rec 8	meiotic recombination protein Rec 8
SAC	spindle assembly checkpoint
Scc1	sister chromatid cohesion protein 1
TRIP13	thyroid hormone receptor interactor 13
